# Group-specific expressions of co-feeding tolerance in bonobos and chimpanzees preclude dichotomous species generalizations

**DOI:** 10.1016/j.isci.2023.108528

**Published:** 2023-11-23

**Authors:** Edwin J.C. van Leeuwen, Nicky Staes, Jake S. Brooker, Stephanie Kordon, Suska Nolte, Zanna Clay, Marcel Eens, Jeroen M.G. Stevens

**Affiliations:** 1 Animal Behaviour and Cognition, Department of Biology, Utrecht University, Padualaan 8, Utrecht 3584 CA, the Netherlands; 2 Behavioural Ecology and Ecophysiology Group, Department of Biology, University of Antwerp, Universiteitsplein 1, 2610 Wilrijk, Belgium; 3 Centre for Research and Conservation, Royal Zoological Society of Antwerp, Koningin Astridplein 26, 2018 Antwerp, Belgium; 4 Department for Comparative Cultural Psychology, Max Planck Institute for Evolutionary Anthropology, Deutscher Platz 6, 04103 Leipzig, Germany; 5 Psychology Department, Durham University, South Road, Durham DH1 3LE, UK; 6 SALTO Agro- and Biotechnology, Odisee University College, Hospitaalstraat 23, 9100 Sint Niklaas, Belgium

**Keywords:** Biological sciences, Zoology, Ethology

## Abstract

Bonobos are typically portrayed as more socially tolerant than chimpanzees, yet the current evidence supporting such a species-level categorization is equivocal. Here, we used validated group-level co-feeding assays to systematically test expressions of social tolerance in sixteen groups of zoo- and sanctuary-housed bonobos and chimpanzees. We found that co-feeding tolerance substantially overlaps between the species, thus precluding categorical inference at the species level. Instead, marked differences were observed between groups, with some bonobo communities exhibiting higher social tolerance than chimpanzee communities, and vice versa. Moreover, considerable intergroup variation was found within species living in the same environment, which attests to *Pan*’s behavioral flexibility. Lastly, chimpanzees showed more tolerance in male-skewed communities, whereas bonobos responded less pronounced to sex-ratio variation. We conclude that the pervasive dichotomy between the tolerant bonobo and the belligerent chimpanzee requires quantitative nuance, and that accurate phylogenetic tracing of (human) social behavior warrants estimations of intraspecific group variation.

## Highlights

• Bonobos and chimpanzees overlap in their levels of co-feeding tolerance
• Group identity is a stronger predictor of co-feeding tolerance than species
• Chimpanzees’ tolerance is positively correlated with male ratio in the group
• Group-specific social dynamics warrant more attention in the *Pan* species

## Graphical abstract

**Figure:**
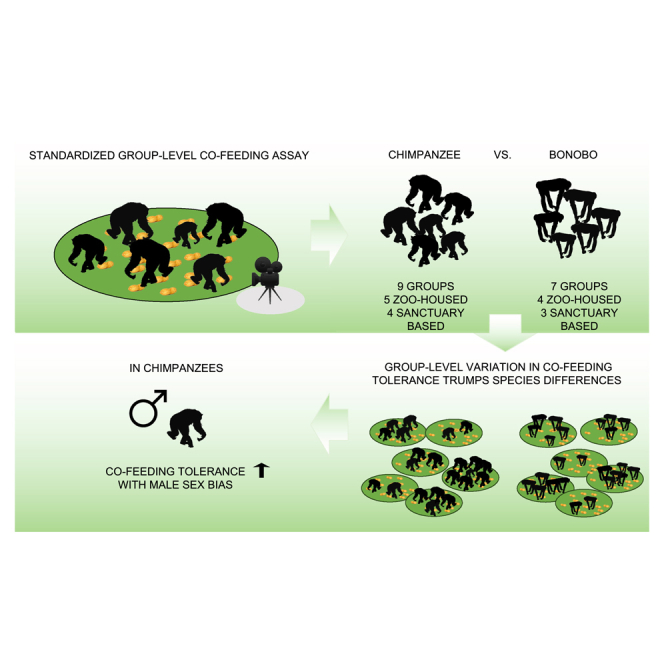

## Introduction

Social tolerance—defined as the propensity to be in enduring close proximity to conspecifics in the presence of valuable resources^1^^,^^2^—lies at the heart of many fitness-affecting behaviors in both humans and other animals. For instance, social tolerance allows for cooperative activities like foraging or predator defense and forms a prerequisite for the transmission of information which in turn enables the adoption of beneficial behavior without the risk inherent to self-exploration.^3^^,^^4^^,^^5^^,^^6^^,^^7^ As such, high levels of social tolerance have been suggested to be a defining characteristic of the human species, to the extent that humans have been depicted as “ultra-social”^8^ and “hyper-cooperative.”^9^ Considering chimpanzees’ (*Pan troglodytes*) and bonobos’ (*Pan paniscus*) close phylogenetic relatedness to humans and each other,^10^ one could expect to find considerable behavioral similarities with humans and between these sibling species. Yet, typically, behavioral *contrasts* between the species are highlighted, with bonobos being portrayed as more peaceful and empathic than chimpanzees.^11^^,^^12^^,^^13^^,^^14^ For instance, while chimpanzees sometimes engage in lethal intergroup aggression and coalitionary killing, such acts of fatal aggression have not been reported for bonobos, whereas in fact, wild bonobo intergroup encounters can often be peaceful.^15^^,^^16^^,^^17^^,^^18^ While some have questioned this apparent dichotomy between the two *Pan* species,^19^^,^^20^ most studies focus on their behavioral differences rather than addressing the ranges of behavioral overlap.^21^^,^^22^

In the realm of social tolerance, the dichotomy between bonobos and chimpanzees has been explicitly postulated and documented. Social tolerance is thought to enable bonobos to peacefully co-feed and cooperate,^21^^,^^22^^,^^23^^,^^24^^,^^25^^,^^26^^,^^27^ while chimpanzees’ *in*tolerance prevents fruitful interactions and cooperation.^6^^,^^14^^,^^28^^,^^29^ However, these postulations are biased given that many studies compare single groups of the two species to draw general conclusions about interspecies differences and their impact on our understanding of human evolution.^19^^,^^30^ To identify relevant predictors of *Pan* socio-dynamics and facilitate our understanding of the evolutionary origins of humans’ ultra-social nature,^8^ systematic comparisons between our two closest living relatives at the group- rather than species-level are needed.^30^^,^^31^^,^^32^^,^^33^

In this study, we test a large sample of independent *Pan* groups to date (n = 16 groups: 7 groups of *Pan paniscus* and 9 groups of *Pan troglodytes*; n = 225 apes) for their group-level expressions of co-feeding tolerance using previously validated experimental assays.^34^^,^^35^^,^^36^^,^^37^ We compare several groups of each species in two different environments: zoos and sanctuaries. Traditionally, comparative research on apes has occurred in zoos where conditions are less natural yet arguably more similar between the two species than in the wild. For instance, across zoo settings, there may be more similarity in diet, food abundance, predation risk (absent), and territory size than in the wild. In sanctuaries, husbandry conditions can be considered less artificial compared to zoo conditions, as the apes typically live in larger groups, in more naturalistic and larger enclosures. Moreover, often several groups of the same species can be studied in the same location under similar within-institution husbandry management.^38^ Apes may differ behaviorally between captive and sanctuary settings due to typically more natural living conditions and less visitor effects in sanctuaries compared to zoos.^38^ Hence, both settings are complementary relevant to assess species and/or group variation in co-feeding tolerance.

For our study, we focus on two behavioral measures of co-feeding tolerance that can be experimentally tested at the group level in a standardized fashion: (1) the proportion of group members co-feeding in a pre-defined food zone in which the space measurements are controlled for group size^3^ and, (2) the temporal fluctuation of this proportion, given that resource availability and related levels of competition are influenced by the co-feeding dynamics in previous time points.^37^ Where the first measure reflects an overall level of co-feeding tolerance, the second measure captures the speed by which group members choose to abandon the competitive context. We test the hypothesis that co-feeding tolerance in bonobos and chimpanzees does not differ at the species level, but rather at the group level. To identify possible drivers of group-level variation in co-feeding tolerance, we assessed the influences of known determinants of sociality in primates, namely group size, genetic relatedness, and group-averaged age.^39^^,^^40^ Lastly, we tested the effect of the groups’ male/female ratio on their respective co-feeding tolerance, as bonobos are matriarchal while chimpanzees are patriarchal.^41^

## Results

### Between-species variation

**Figure 1:**
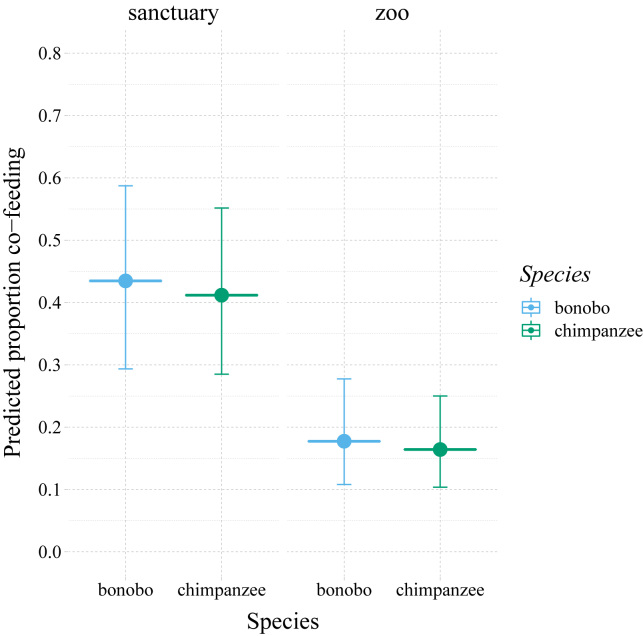
Co-feeding for the *Pan* species across the different settings Bonobos and chimpanzees (x axis) do not differ in their co-feeding propensities measured as the proportion of group members present during a standardized co-feeding experiment. The left panel depicts the sanctuary apes, the right panel the zoo apes. Filled circles represent the predicted values (y axis); the error bars represent the 95% confidence intervals.

Bonobos and chimpanzees did not differ in their propensity to tolerate each other around food resources (likelihood ratio test species: χ^2^ = 0.057, df = 1, p = 0.81; mean ± SD proportion in resource zone: bonobos = 0.34 ± 0.17; chimpanzees = 0.34 ± 0.23; estimate ±SE = −0.07 ± 0.29; 95% CI: −0.67– 0.53; Figure 1).

**Figure 2:**
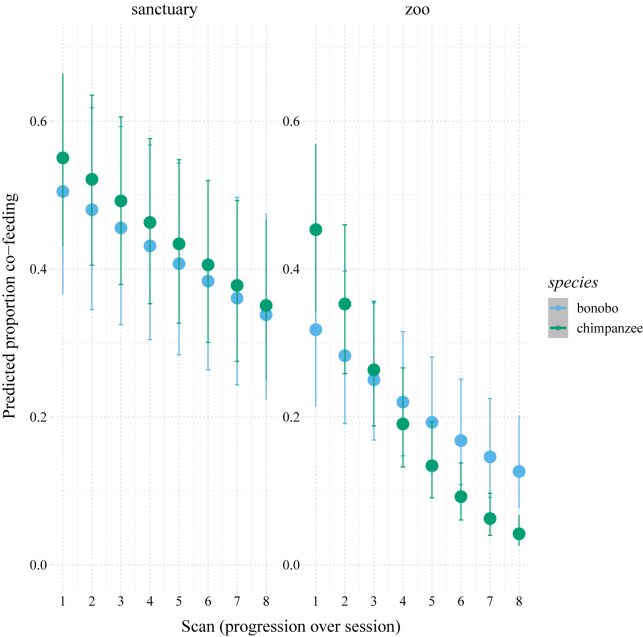
Co-feeding progression over time for both *Pan* species In zoo settings (right panel), bonobos and chimpanzees differ in their co-feeding strategies (y axis) when resources are depleting over experimental time (x axis), whereas in sanctuary settings (left panel), their strategies do not obviously differ. Filled circles represent the predicted values; the error bars represent the 95% confidence intervals.

With increasing resource depletion (represented by scan number), however, their co-feeding tolerance progressed in different ways (LRT species|scan interaction: χ^2^ = 12.49, df = 1, p < 0.001), which, upon visual inspection, seemed mainly due to differences between the species in the zoo settings rather than in the sanctuary settings (Figure 2). Lastly, co-feeding tolerance was generally higher for the *Pan* species in the sanctuaries compared to the zoo setting (LRT: χ^2^ = 10.97, df = 1, p < 0.001; estimate ±SE = −1.21 ± 0.30; 95% CI: −1.85 to −0.57), although this effect was not obviously different for the two species (see Figure 1). Note, here, that due to logistical constraints, different versions of the co-feeding tolerance test were used in the zoos (pasta plot) compared to the sanctuaries (peanut swing),^37^ which precludes a direct interpretation in terms of *Pan* behaving differently in the respective settings (see STAR methods).

### Between-group variation

**Figure 3:**
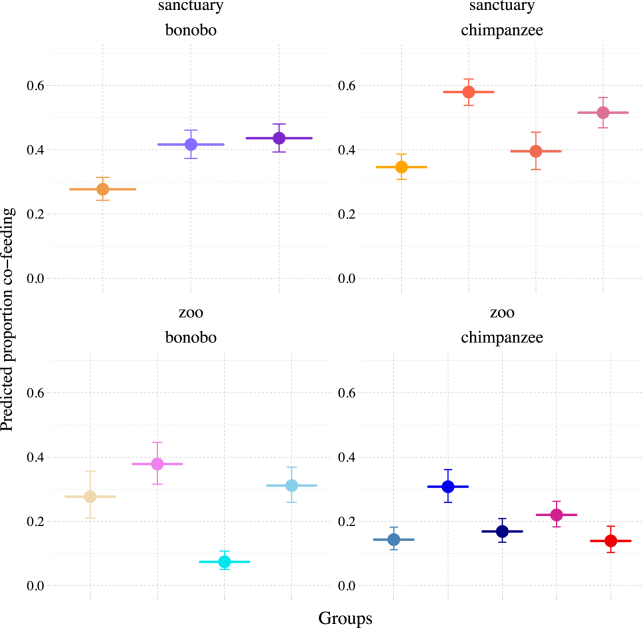
Predicted co-feeding proportions per group Co-feeding tolerance is group specific in the *Pan* species in sanctuary settings (upper panel) and zoo settings (lower panel). Bonobos are depicted in the left panels, chimpanzees on the right. Filled circles represent the predicted values (y axis); the error bars represent the 95% confidence intervals.

**Figure 4:**
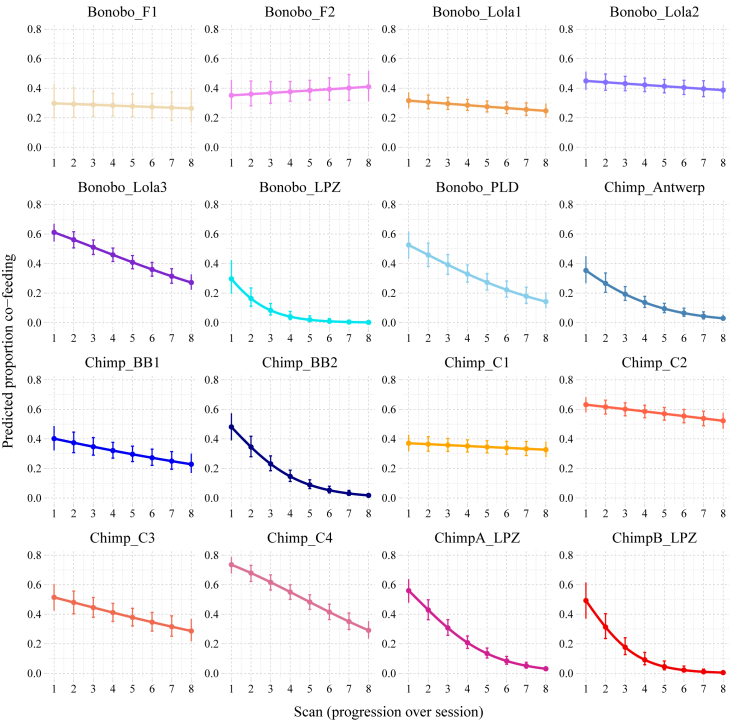
Predicted co-feeding progressions over time per group *Pan* groups differ substantially in their co-feeding strategies (y axis) when resources are depleting over experimental time (x axis). Some groups stably co-fed with a relatively low proportion of group members in the resource zone until the resources were depleted (e.g., Bonobo_F1 and Chimp_C1), whereas others co-fed with almost the entire group from the beginning after which the proportion decreased steadily (e.g., Chimp_C4). Filled circles represent the predicted values (y axis); the error bars represent the 95% confidence intervals. The colors are group specific and match the colors for the same groups in Figure 3.

At the group level, the *Pan* communities differed substantially from each other in their co-feeding propensities (LRT on “Group”: χ^2^ = 81.53, df = 1, p < 0.001; Figure 3). Similarly, the progression of co-feeding tolerance over experimental time was highly group specific (LRT group|scan interaction: χ^2^ = 226.45, df = 15, p < 0.001; Figure 4). Importantly, in these group-level models (see STAR methods), “species” did not have any additional explanatory power (χ^2^ = 0, df = 1, p = 1).

**Figure 5:**
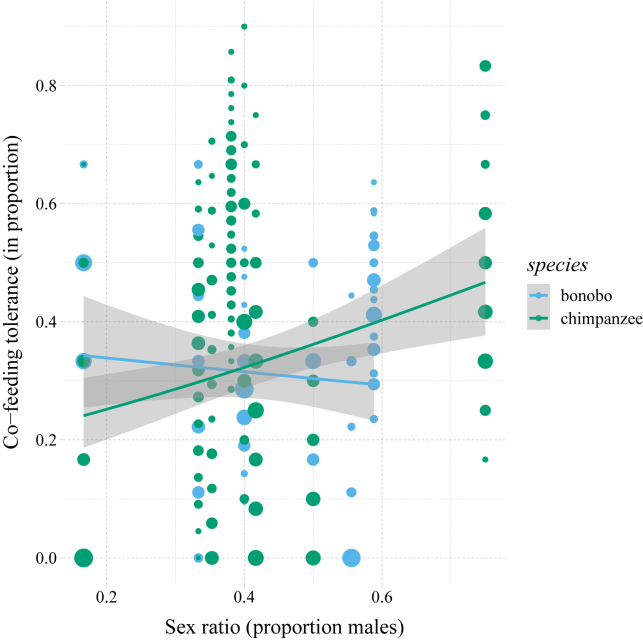
Co-feeding proportions for bonobos and chimpanzees as a function of the group-level sex ratio Co-feeding tolerance (*y* axis) is differently moderated by sex ratio (x axis) for bonobos and chimpanzees. Circles scale with the number of observations, the blue lines represent the model regression trends, and the shaded area around the lines the 95% confidence intervals.

Furthermore, while controlling for the following group-level metrics: group size, average relatedness, age, and sex ratio (see below), “group” remained a highly important factor explaining variation in co-feeding tolerance (χ^2^ = 57.25, df = 1, p < 0.001). The group-level metrics themselves did not significantly affect co-feeding tolerance (all p > 0.14), except for “sex ratio,” which affected co-feeding tolerance differentially for bonobos and chimpanzees (LRT interaction: χ^2^ = 10.34, df = 1, p < 0.002). Co-feeding tolerance increased more for chimpanzees than for bonobos with a higher proportion of males in the group (Figure 5). Caution with interpreting the group-level effects is warranted, however, given the relatively large ranges of respective estimates resulting from the model stability check (Figure S1; see STAR methods).

Table S1 (zoo settings) and Table S2 (sanctuaries) provide statistical details on all group contrasts (separate for the two settings in light of the significant main effect of “setting”). Interestingly, in some facilities and sanctuaries where several *Pan* groups were housed, we found substantial within-species differences in co-feeding tolerance despite their similar facility setups, husbandry styles, and other potentially latent determinants like the weather and (the absence of) visitors (see supplementary materials).

## Discussion

By systematically comparing a relatively large sample of independent *Pan* groups in two settings (zoos and sanctuaries), we show that co-feeding tolerance is not universally more pronounced in bonobos compared to chimpanzees,^14^ but is in fact a group-specific characteristic in the *Pan* species. The *Pan* groups in our sample differed substantially with respect to how many group members could benefit from food resources simultaneously. Moreover, groups differed in their strategies of co-feeding: whereas in some groups the apes were only together for one or two scans, others were characterized by continuous co-feeding until the food was depleted. These results put earlier findings on single (or fewer) groups into perspective: bonobos as a species are neither more tolerant with respect to co-feeding than chimpanzees (^14^ cf; ^42^^,^^43^), nor does the opposite view hold.^35^^,^^44^ As such, this study resonates with the recently reiterated message that intraspecific variation in social behavior needs to be accounted for when drawing inferences about species-level characteristics.^30^^,^^33^^,^^36^^,^^45^^,^^46^^,^^47^ Here, we explicitly note that we have tested captive *Pan* populations (in zoos and sanctuaries) and that we do not know to what extent our findings would generalize to their wild counterparts. A recent study in lemurs^48^ found higher levels of co-feeding tolerance in captive versus wild groups, whereas another study reported the opposite pattern for common marmosets.^49^ Given that a complex interplay between ecological and social conditions (e.g., food availability and magnitudes of interdependence^50^^,^^51^), including potential cultural differences,^52^^,^^53^ likely shapes wild *Pan*’s expressions of co-feeding tolerance, this question remains outstanding for future research.

To investigate which mechanisms might be involved in creating the observed group differences in co-feeding tolerance, we assessed the effect of a group-level index central to the social organization of the *Pan* species: sex ratio.^41^ The proportion of males in the group was more positively related to co-feeding tolerance in chimpanzees than bonobos, which may be explained by the male-biased dominance system in chimpanzees.^54^ Overall, though, there may be more socio-demographic factors (e.g., number of immatures) that could affect co-feeding tolerance in the *Pan* species.^55^ However, the sample size at the group level (n = 16) did not allow for incorporating more variables to estimate, and thus for identifying robust group-level determinants of co-feeding tolerance, which is a key avenue to explore in the future.^30^ We also note that our conclusions are limited to the co-feeding contexts created by our paradigms—our findings neither negate nor preclude the possibility of species differences in other measures of tolerance, e.g., in the context of intergroup encounters.^16^^,^^18^ As such, our results are specifically relevant for revealing the breadth of co-feeding tolerance in bonobos and chimpanzees, including their overlap,^35^ which warrants further systematic studies in other domains of social tolerance.^3^

At the proximate level, our findings are relevant for understanding the possible causes of behavior in the *Pan* species more generally. If co-feeding tolerance reflects sociality in terms of daily interaction patterns more generally (see Cronin et al. and van Leeuwen et al.^34^^,^^36^), then other social behavior like social learning, cooperation, or resource sharing could substantially correlate with tolerance (e.g., see van Boekholt et al. and Cantor and Whitehead^56^^,^^57^). In agreement with this, a recent study found marked differences in expressions of prosociality between groups of chimpanzees living in the same sanctuary, with the groups highest in prosociality also being the highest in co-feeding tolerance.^36^ Similarly, in Japanese macaques, substantial group differences in social tolerance were found,^58^ which positively correlated with cooperative success on an experimental task.^5^ Such intergroup variation in sociality has been documented in other species as well, for instance, vervet monkeys,^33^^,^^59^ meerkats,^60^ and whales.^61^ These indications of inter-group variation in social behavior caution against the use of single group studies for broad species inference.^30^ For instance, finding that one group of chimpanzees behaves relatively indifferent to benefitting others in a group context,^9^ bears little relevance to characterizing their species-specific ways of behaving when other groups seem relatively prosocial with a similar experimental design.^36^ Rather, the aggregation of these (independent) tests could cumulatively paint a picture of the species-specific *range* of behaving (also see DeTroy et al.^55^), which in turn could be used to compare against ranges of other species, like in the current study.

At the ultimate level, our findings point toward group-specific social dynamics as a main determinant of social tolerance. The proportion of group members that could benefit from the valuable resources, including their progression over time, varied substantially from group to group, which indicates that in both bonobos and chimpanzees the local group climate determines in large part how resources will distribute over the group members.^55^ Tolerance is likely to be a result of group living,^62^ with the magnitude of its expression being shaped by the fitness benefits of the behaviors it facilitates, like cooperation^16^ and social learning.^4^ Such feedback loops may be influenced by (the threat of) intergroup competition, spurring increased social tolerance, affiliation, and cooperation *within* groups.^63^^,^^64^ As such, it would have been valid to assume that chimpanzees and not bonobos show higher levels of social tolerance, given that their evolutionary environment of adaptation most likely comprised higher levels of intergroup conflict.^16^ While such intergroup encounters turn out to be markedly more peaceful in bonobos compared to chimpanzees,^12^^,^^16^ the findings of the current study reveal similar underlying propensities with respect to tolerance around valuable resources when environmental conditions (e.g., food availability and distribution) and possible effects of group-level metrics (e.g., group size, sex ratio)^65^ are controlled for. This inconsistency may further attest to *Pan*’s large behavioral flexibility to adapt to various environmental and social circumstances.^66^^,^^67^

Taken together, the current study shows that bonobos and chimpanzees in zoo and sanctuary settings behave tolerantly toward group members in a group-specific, rather than a species-specific manner. Future multi-group research is warranted to test whether this finding extends beyond tolerance in the competitive context of limited food resources, as measured in the current study, to other contexts in which tolerance could play a pivotal role, like during daily interaction patterns (e.g., for social learning, cooperation, or reconciliation) and/or intergroup encounters. In conjunction, such assessments can identify the scope of adaptive potential within the panins and may provide leads as to the selection pressures shaping the form and function of group-level interaction styles for panins and possibly also for hominins.

## STAR★Methods

### Key resources table

**Table:** 

REAGENT or RESOURCE	SOURCE	IDENTIFIER
**Experimental models: Organisms/strains**		

*Pan paniscus*	Frankfurt Zoo, Planckendael Zoo, Leipzig Zoo, Lola Ya Bonobo	
*Pan troglodytes*	Leipzig Zoo, Antwerp Zoo, Beekse Bergen Zoo, Chimfunshi Wildlife Orphanage	

**Software and algorithms**		

R	R Core Team, 2020	http://www.r-project.org/
Lme4 package in R (v. 4.1.0)	Bates, D., Mächler, M., Bolker, B. & Walker, S.	https://cran.r-project.org/web/packages/lme4/index.html

### Resource availability

#### Lead contact

Further information and requests for resources should be directed to and will be fulfilled by the lead contact: Edwin J. C. van Leeuwen (e.j.c.vanleeuwen@uu.nl).

#### Materials availability

This study did not generate new unique reagents.

#### Data and code availability

(1) All data used in this study are available at a public repository (https://doi.org/10.5061/dryad.m0cfxpp23).
(2) This paper does not report original code.
(3) Any additional information required to reanalyze the data reported in this paper is available from the lead contact upon request.

### Experimental model and study participant details

We studied 16 groups of great apes (N_bonobo_ = 7; N_chimpanzee_ = 9) totalling 225 *Pan* individuals (N_bonobo_ = 82 (46f/36m); N_chimpanzee_ = 143 (85f/58m)) across 7 independent sites (5 zoological institutions, 2 African sanctuaries) between July 2018 – September 2019. The zoological institutions were Frankfurt Zoo (two groups of bonobos), Planckendael Zoo (1 group of bonobos), Leipzig Zoo (1 group of bonobos; 2 groups of chimpanzees), Antwerp Zoo (1 group of chimpanzees), Beekse Bergen Zoo (2 groups of chimpanzees). The sanctuaries were Lola Ya Bonobo (3 groups of bonobos), and Chimfunshi Wildlife Orphanage (4 groups of chimpanzees). Tables S3 and S4 provide demographic information on the study subjects and group-level indices.

### Method details

#### Experimental measures

We measured two components of co-feeding tolerance as a proxy for social tolerance,^3^ because they could be assessed experimentally and in a standardized way across groups.

To do so, we administered two established group-level co-feeding assays: the “peanut swing” in the great ape sanctuaries,^34^ and the “peanut plot”^35^ in the zoo settings (Figure S2). Both assays comprise linear adjustments for group size such that larger groups receive more resources across a larger resource zone (see^34^^,^^35^^,^^37^). Owing to practical constraints, however, the original peanut swing assay could not be administered in the zoo settings – hence, the peanut plot was devised (see^35^). In a recent systematic investigation, the peanut swing and peanut plot assays produced very similar co-feeding dynamics in chimpanzees.^37^ However, given that some great apes in the zoo settings were known to show allergic responses to peanuts, we adapted the peanut plot in all zoos by replacing the peanuts for cooked pasta (*Penne rigate*).

The peanut swing consisted of a sliced-through (in length) bamboo trunk in which a predetermined number of peanuts (12/ape aged ≥ 3 years) was distributed across a predetermined length (20 cm/ape aged ≥ 3 years). The peanuts were deployed in the enclosure by forcefully protruding the swing toward the fence, causing the peanuts to spread in the enclosure over a ±1 m width. Before the peanuts were thrown in the enclosure, the apes were attracted to the fence by shaking a bucket with peanuts while indicating vocally that the peanut swing session was about to start. Sessions would only start when at least 90% of the subjects were visibly accounted for (with the remaining subjects typically being in close but hidden vicinity). The mean number of test sessions per group was 11.6 (range 8–13 sessions).

The pasta plot was identical to the peanut swing, except for the food resource, the means by which the food was deployed in the enclosure, and the onset. The pasta was distributed in the outdoor enclosure in a rectangle on the ground (cleared of grass) with a predetermined length (20 cm/ape aged ≥3 years) and 1 m width, while the apes were in the indoor holding facility for their mid-day feeding. To create group-wide attention to the experiment, similar to the bucket shaking and vocal calling in the peanut swing, the pasta was shown to the apes in the indoor facility before the pasta was placed, and the apes were also able to witness through windows how and where the pasta was put in the outside enclosure. When the apes entered the outside enclosure, they would directly find the pasta, after which the session began (T_0_ = arrival of the first ape in the resource zone). In contrast to the peanut swing, during the pasta plot experiment, the apes could not immediately be made aware of the fact that an experiment was about the commence. Thus, to familiarize the apes with the procedure of pasta plot sessions and the location of the pasta, we administered three familiarization sessions on three different days before actual test sessions started. During these familiarization sessions, the experimenter followed the same procedures as described above but with no behavioral recording. Only the test sessions were included in the analyses. The mean number of test sessions per group was 7.4 (range 5–8 sessions).

#### Experimental procedure and coding

All assays followed the same general procedure. Only one session was administered per group per day. For all sessions, we used a minimum of 1 h latency since the last regular feeding. The sessions were video recorded from two vantage points and started when swinging the peanuts into the enclosure (swing) or opening the doors of the holding facility (plot). T_0_ was determined by the arrival of the first ape in the resource zone, which was defined as the zone in which an ape could access the resource, set to 1 m around the border of the peanut/pasta rectangles. Each session consisted of 8 scan points with 15s intervals (i.e., 2 min in total), starting at T_15_ (in sec). This was roughly the time the chimpanzees needed to consume all peanuts in the original peanut swing experiment.^34^ For each scan, the number of apes present in the resource zone was scored from video. By definition, these scans represented our temporal measure of co-feeding tolerance (i.e., the slopes over time). To gauge inter-rater reliability, the lead author (EJCvL) coded one random session from all study groups that were tested and coded by others (n = 12 groups, n = 7 experimenters), and calculated intraclass correlations respectively. Agreement on the number of apes in the resource zone was considered “excellent” (mean ICC = 0.940; range = 0.708–1).^68^

### Quantification and statistical analysis

We modeled co-feeding tolerance (number of individuals in the resource zone/number of individuals *not* in the resource zone) at the scan level using Generalized Linear Mixed Models with Binomial error distribution and logit link function.^69^ We chose this approach over modeling the data with a beta distribution because the response variable (i.e., individuals/group) can take only discrete proportions, whereas the beta distribution assumes the possibility of a continuous range of values between 0 and 1.

The full model (model 1) consisted of the fixed effects “species”, “scan number” (denoting the progression of co-feeding tolerance over time within the session, i.e., with increasing resource depletion), “sex ratio” (proportion of males in the group) and “setting” (zoo or sanctuary), including the two-way interactions between species and scan number (to also test for species-level variation in co-feeding tolerance with increasing resource depletion) and species and sex ratio.^41^ The random effects structure consisted of the intercept of “group”, and “session” nested in group including the random slope of “scan number” (*z*-transformed) therein, excluding the interaction between intercept and slope.^70^ Given that all sessions took place in the apes’ outdoor enclosures, which coincided with relatively comfortable weather conditions, we did not test for effects of housing and/or climatological factors.

Before assessing the impact of single parameters, a full-null model comparisons was conducted using a likelihood ratio test (LRT:^71^^,^^72^) with the null model comprising only “setting” and the random effects structure (χ^2^ = 117.2, df = 5, p < 0.001). We chose to use “setting” as control variable, because it denotes not only variation in test location, but also experimental procedure and food resource (see STAR methods). Yet, to enable inspection of the influence of this combined variable on co-feeding tolerance,^37^ we interpret the main effect and visualize the results separately for each setting. To test for species differences, we compared the full model to a reduced model with the term “species” removed using an LRT. To test for group differences, we compared the full model with a model excluding the random effect of “group” using LRT.

In a second model (model 2), we included the group-level metrics known to affect primates’ social behavior: group size, average relatedness (proportion maternally related dyads), age (group average), and sex ratio (proportion of males) in interaction with species.^41^ Crucially, we again tested for the effect of “group” (by means of a model comparison with a LRT^72^) to investigate the extent to which potential group differences in co-feeding tolerance observed in model 1 were explained by the group-level metrics. Given that these group-level metrics contained less variation (n = 16 groups) than the modeled co-feeding data (n = 1,176 scan points), here, we tested for estimate stability by iteratively running the model with a leave-one-out procedure (with replacement).

To test whether groups differed in co-feeding tolerance with increasing resource depletion, we ran an additional model (model 3) with “group” as fixed effect in interaction with “scan”. In this model, “species” and “setting” were moved to the random effects structure, because species is nested within group, which causes the model to be rank-deficient and automatically dropping meaningful coefficients. Here, to validate model 1, we again tested for a species effect by comparing the model with and without the random effect of “species”.

Parameter inspection was done with the “drop1” function (LRT) and, for testing contrasts between the groups, the *emmeans* R package.^73^ The full models were not overdispersed (model 1: p = 1; model 2: p = 1; model 3: p = 1), but mildly underdispersed (dispersion parameter = 0.54, 0.59, and 0.70, respectively), which is considered harmless for drawing inference, as it tends to lead to conservative rather than anti-conservative results.

All models were fitted in R (v 4.0.2^74^) using the function “glmer” of the R package *lme4*.^75^ p-values <0.05 were considered significant.

## Supplemental information

Document S1. Figures S1, S2, Tables S1, S2, and S4

Table S3. Study subjects including demographics and group identity, related to STAR methods
