# Supplementary material for: Group-specific expressions of co-feeding tolerance in bonobos and chimpanzees preclude dichotomous species generalizations
Source: iScience. 2023 Nov 23;26(12):108528. doi: 10.1016/j.isci.2023.108528 (PMC10746535; doi:10.1016/j.isci.2023.108528)
Supplement: Document S1. Figures S1, S2, Tables S1, S2, and S4 [file mmc1.pdf]

iScience, Volume *26*

Supplemental information

Group-specific expressions of co-feeding tolerance in bonobos and chimpanzees preclude dichotomous species generalizations

Edwin J.C. van Leeuwen, Nicky Staes, Jake S. Brooker, Stephanie Kordon, Suska Nolte, Zanna Clay, Marcel Eens, and Jeroen M.G. Stevens

Supplementary materials for:

The Supplementary Materials include:

Methods
Results
Figures and Tables

Methods

We sampled 16 captive and semi-wild *Pan* groups (n=225 individuals) to assess inter- and intraspecific variation in group-level social tolerance as measured with standardized co-feeding paradigms (Figure S2). Here, the focus was not on individual (e.g., personality[1,2]) or dyadic (e.g., social bonds[3,4]) differences, but on a group-level expression of a social style (like dominance: e.g.,[5,6]) in which the respective individuals reside and "operate". These group-level climates form the substrate from which individuals navigate their social and non-social worlds, and as such form an important factor in understanding (social) behaviour more generally[7–9]. For clarity and readability, we name the group identifies in Table S1 and S2, in which we provide the separate comparative contrasts separate for zoo- and sanctuary housed groups (see below).

For calculating the number of food items to be distributed in the resource zone, we treat the young individuals (as of 3-yrs of age) the same as the adults in our experimental data coding for the reason that they are all independent foragers capable of being in the resource zone independently and eating the peanuts/pasta independently. Before the age of 3-yrs, there is a high chance that the youngsters are clinging to their mothers and/or refrain from participating altogether. Given that we cannot operationalize a behavioural indicator that is both clearly identifiable and a reliable reflection of individuals being able to participate independently in a feeding competition, we have used the same age-related cut-off for all groups to facilitate systematic comparisons. Furthermore, we are working under the assumption that even though young individuals eat less than adults, neither youngsters nor adults will become satiated from eating a handful (i.e., 12 pieces) of peanuts/pasta.

Results

Within facility intra- and interspecific variation

Within the data set, there are several *Pan* groups included from the same facilities. A comparison of within-facility *Pan* groups is of particular relevance for the study of intra- and interspecific variation because of the similarity of conditions (i.e., physical climate, husbandry protocols, space availability, housing protocols, diet, etc.). As such, in these comparisons, we can plausibly assume less variation attributable to external factors and consequently more variation attributable to within-group social dynamics.

<u>Bonobos</u>: two facilities housed multiple groups of bonobos: Frankfurt Zoo (Germany) and Lola Ya Bonobo (The Democratic Republic of Congo). Within Frankfurt, the two groups of bonobos expressed similar levels of co-feeding tolerance ($z = 1.89$, $p = 0.62$; mean proportion of the group in the resource zone ± SD "Group 1" = 0.28 ± 0.13; "Group 2" = 0.38 ± 0.14; see Table S1). The three groups at Lola Ya Bonobo, however, differed substantially in their expressed magnitudes of co-feeding tolerance. More specifically, two relatively tolerant groups expressed higher levels of co-feeding tolerance than one relatively intolerant group ("Group 1" vs "Group 2": $z = 4.53$, $p = 0.001$; "Group 1" vs "Group 3": $z = 5.10$, $p < 0.001$; "Group 2" vs "Group 3": $z = 0.52$, $p = 0.999$; mean ± SD "Group 1" = 0.28 ± 0.07; "Group 2" = 0.42 ± 0.09; "Group 3" = 0.44 ± 0.15; see Table S2).

<u>Chimpanzees</u>: three facilities housed multiple groups of chimpanzees: Beekse Bergen (the Netherlands), Leipzig Zoo (Germany), and the Chimfunshi Wildlife Orphanage Trust (CWOT; Zambia). Within Beekse Bergen, the two groups of chimpanzees expressed substantially different levels of co-feeding tolerance ($z = 5.34$, $p < 0.001$; mean proportion of the group in the resource zone ± SD "Group 1" = 0.32 ± 0.12; "Group 2" = 0.18 ± 0.20; see

Table S1). At Leipzig Zoo, the two groups of chimpanzees did not significantly differ in their expression of co-feeding tolerance ($z = 2.64$, $p = 0.16$; mean ± SD “Group 1” = 0.32 ± 0.12; “Group 2” = 0.18 ± 0.20; Table S1). The four groups at CWOT did again substantially differ in their levels of co-feeding tolerance (mean ± SD “Group 1” = 0.35 ± 0.14; “Group 2” = 0.58 ± 0.12; “Group 3” = 0.40 ± 0.17; “Group 4” = 0.51 ± 0.20; see Table S2). It is noteworthy that the differences in co-feeding tolerance at CWOT corroborate previously reported differences across the same study populations[10–12], indicating that co-feeding tolerance in chimpanzees may be a temporally stable group-specific trait despite changes in group composition (over a time period of at least 8 years, also see[10], but compare:[13]).

Bonobos vs chimpanzees: one facility (LPZ) housed one bonobo group and two chimpanzee groups. The most tolerant chimpanzee group (mean ± SD = 0.23 ± 0.21) was significantly more tolerant than the bonobo group (mean ± SD = 0.08 ± 0.13; $z = 4.84$, $p < 0.001$), whereas the least tolerant chimpanzee group (mean ± SD = 0.15 ± 0.18) expressed statistically similar levels of co-feeding tolerance as the bonobo group ($z = 2.29$, $p = 0.35$; see Table S1).

Figures and Tables

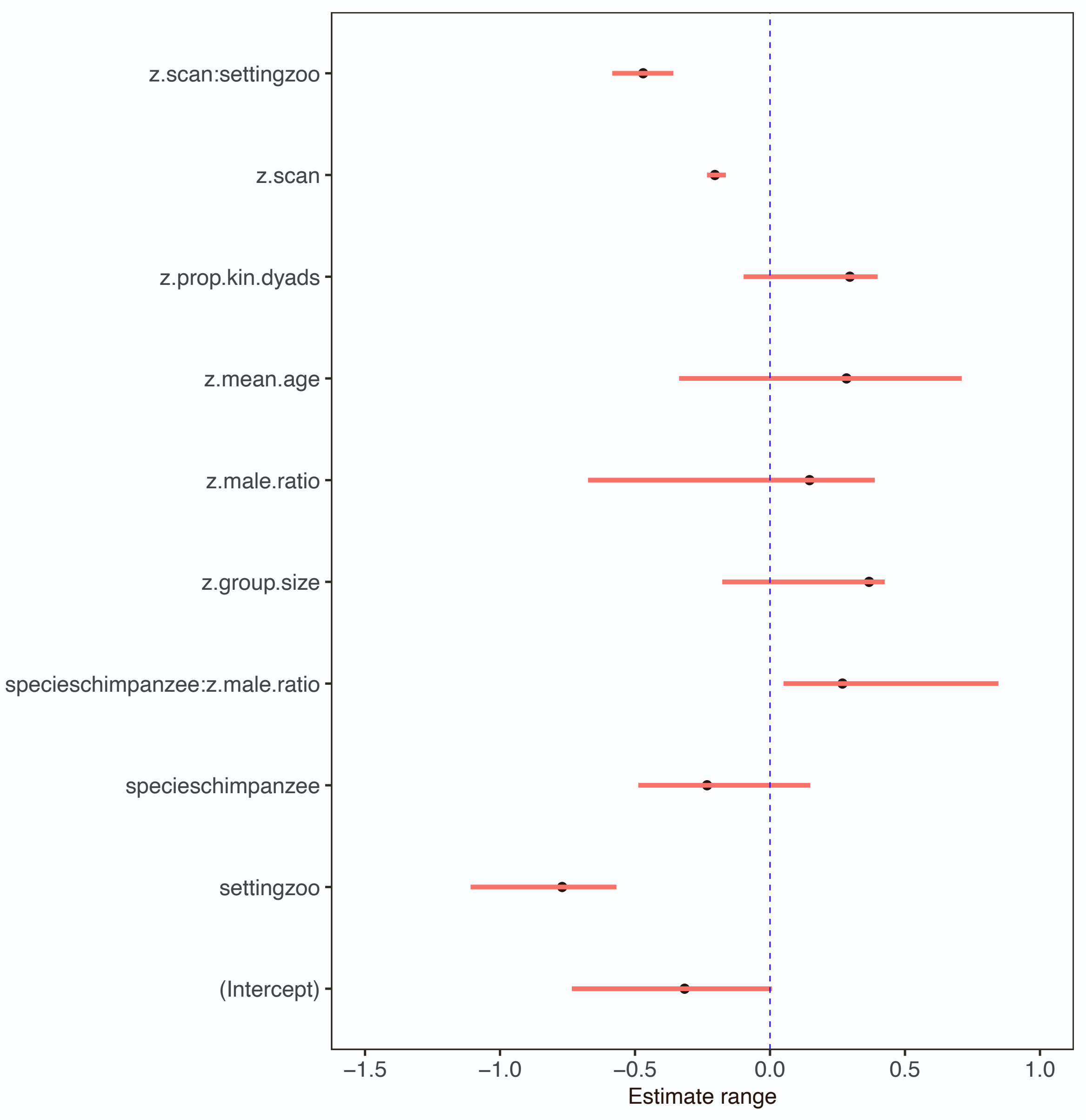

Figure S1. Model stability assessment (related to Figure 5). Model stability assessment via sequential omission (with replacement) of groups from the group-level analysis including the group-level metrics. Parameters (across the y-axis) are depicted with their respective estimate ranges (x-axis). To obtain these model estimate ranges, we re-ran the model with each time one group omitted (so with group N = 15). As such, we obtained multiple estimates for each assessed variable (y-axis). The range of these estimates is taken as a reflection of how robust the estimate of the full model (group N = 16) is against specific group omissions and thus against having specific configurations of groups in our sample.

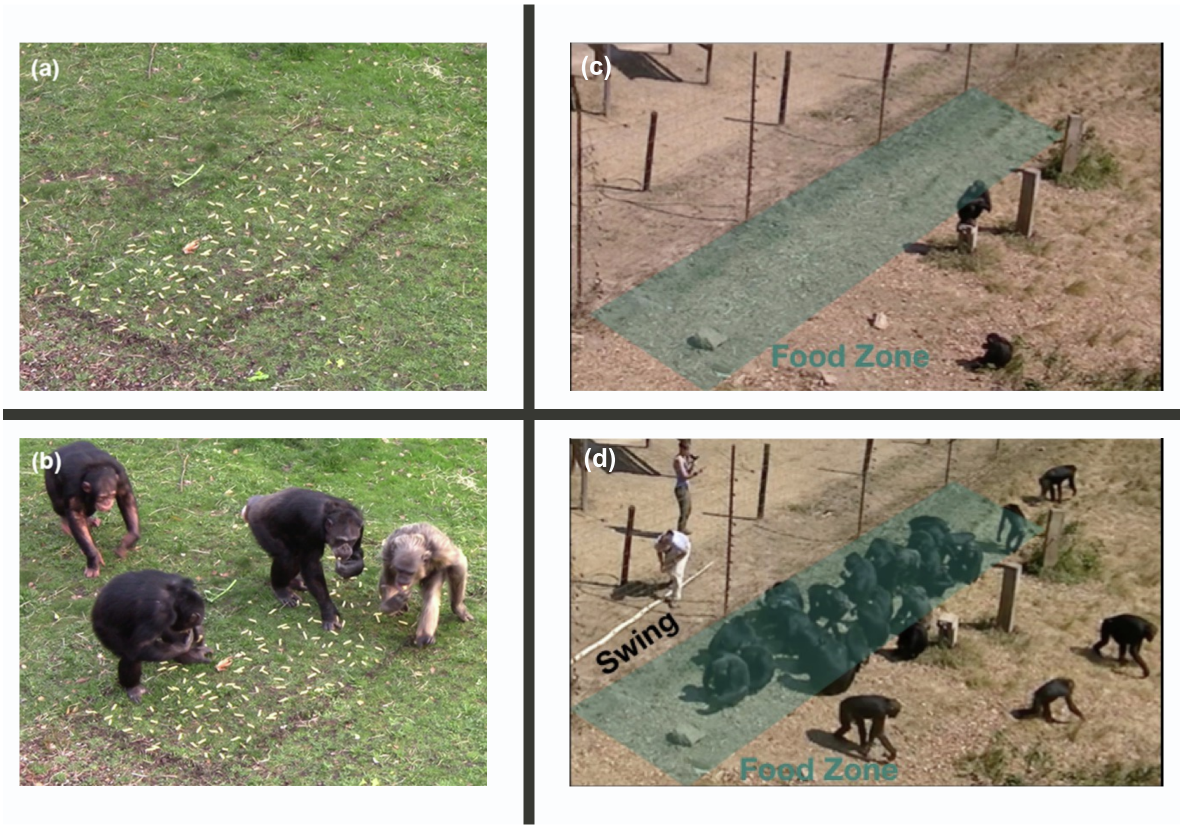

Figure S2. Depiction of experimental designs (related to *Experimental Measures* in Main Text). The two standardized co-feeding tolerance measures: the pasta plot (a-b) as used in the zoo-settings, and the peanut swing (c-d) as used in the sanctuary-settings. Proportional to group size, a pre-determined number of food resources were distributed (b and d) across a pre-determined ground-surface, after which co-feeding was operationalized as the proportion of group members who simultaneously co-fed across experimental time (2 minutes).

Table S1 (related to *2.2 Between-group variation*). Group-level differences in co-feeding tolerance across Zoo-housed *Pan* communities expressed in all possible contrasts.

Group contrast	Estimate	SE	*P* value*
ChimpA_LPZ - Chimp_Antwerp	0.4670	0.221	0.4645
ChimpA_LPZ - Chimp_BB1	-0.7459	0.196	0.0046
ChimpA_LPZ - Chimp_BB2	0.3957	0.216	0.6608
ChimpA_LPZ - Bonobo_LPZ	1.4433	0.298	<.0001
ChimpA_LPZ - ChimpB_LPZ	0.6781	0.257	0.1695
ChimpA_LPZ - Bonobo_F1	-0.6108	0.246	0.2396
ChimpA_LPZ - Bonobo_F2	-1.0859	0.208	<.0001
ChimpA_LPZ - Bonobo_PLD	-0.7099	0.203	0.0141
Chimp_Antwerp - Chimp_BB1	-1.2129	0.218	<.0001
Chimp_Antwerp - Chimp_BB2	-0.0713	0.236	1
Chimp_Antwerp - Bonobo_LPZ	0.9763	0.313	0.047
Chimp_Antwerp - ChimpB_LPZ	0.2111	0.273	0.9976
Chimp_Antwerp - Bonobo_F1	-1.0778	0.263	0.0014
Chimp_Antwerp - Bonobo_F2	-1.5530	0.229	<.0001
Chimp_Antwerp - Bonobo_PLD	-1.1769	0.224	<.0001
Chimp_BB1 - Chimp_BB2	1.1416	0.214	<.0001
Chimp_BB1 - Bonobo_LPZ	2.1892	0.298	<.0001
Chimp_BB1 - ChimpB_LPZ	1.4240	0.256	<.0001
Chimp_BB1 - Bonobo_F1	0.1351	0.241	0.9998
Chimp_BB1 - Bonobo_F2	-0.3400	0.203	0.7628
Chimp_BB1 - Bonobo_PLD	0.0360	0.198	1
Chimp_BB2 - Bonobo_LPZ	1.0476	0.307	0.0189
Chimp_BB2 - ChimpB_LPZ	0.2824	0.268	0.9803
Chimp_BB2 - Bonobo_F1	-1.0065	0.26	0.0035
Chimp_BB2 - Bonobo_F2	-1.4816	0.225	<.0001

Chimp_BB2 - Bonobo_PLD	-1.1056	0.22	<.0001
Bonobo_LPZ - ChimpB_LPZ	-0.7652	0.334	0.3487
Bonobo_LPZ - Bonobo_F1	-2.0541	0.333	<.0001
Bonobo_LPZ - Bonobo_F2	-2.5292	0.306	<.0001
Bonobo_LPZ - Bonobo_PLD	-2.1532	0.302	<.0001
ChimpB_LPZ - Bonobo_F1	-1.2889	0.296	0.0005
ChimpB_LPZ - Bonobo_F2	-1.7640	0.265	<.0001
ChimpB_LPZ - Bonobo_PLD	-1.3880	0.261	<.0001
Bonobo_F1 - Bonobo_F2	-0.4751	0.251	0.6204
Bonobo_F1 - Bonobo_PLD	-0.0991	0.247	1
Bonobo_F2 - Bonobo_PLD	0.3760	0.21	0.6889

*with Tukey adjustment for multiple testing

Table S2 (related to *2.2 Between-group variation*). Group-level differences in co-feeding tolerance across sanctuary-housed *Pan* communities expressed in all possible contrasts.

Group contrast	Estimate	SE	*P* value*
Chimp_C1 - Chimp_C2	-0.9507	0.131	<.0001
Chimp_C1 - Chimp_C3	-0.2075	0.16	0.8531
Chimp_C1 - Chimp_C4	-0.6973	0.138	<.0001
Chimp_C1 - Bonobo_Lola1	0.3152	0.134	0.2171
Chimp_C1 - Bonobo_Lola2	-0.3005	0.135	0.2792
Chimp_C1 - Bonobo_Lola3	-0.3711	0.133	0.0797
Chimp_C2 - Chimp_C3	0.7432	0.159	0.0001
Chimp_C2 - Chimp_C4	0.2534	0.137	0.5102
Chimp_C2 - Bonobo_Lola1	1.2659	0.132	<.0001
Chimp_C2 - Bonobo_Lola2	0.6502	0.133	<.0001
Chimp_C2 - Bonobo_Lola3	0.5797	0.132	0.0002
Chimp_C3 - Chimp_C4	-0.4898	0.165	0.0461

Chimp_C3 - Bonobo_Lola1	0.5227	0.161	0.0199
Chimp_C3 - Bonobo_Lola2	-0.0930	0.162	0.9975
Chimp_C3 - Bonobo_Lola3	-0.1635	0.161	0.9501
Chimp_C4 - Bonobo_Lola1	1.0125	0.139	<.0001
Chimp_C4 - Bonobo_Lola2	0.3968	0.14	0.0699
Chimp_C4 - Bonobo_Lola3	0.3263	0.139	0.222
Bonobo_Lola1 - Bonobo_Lola2	-0.6157	0.136	0.0001
Bonobo_Lola1 - Bonobo_Lola3	-0.6863	0.135	<.0001
Bonobo_Lola2 - Bonobo_Lola3	-0.0705	0.136	0.9986

*with Tukey adjustment for multiple testing

Table S4 (related to STAR methods). Demographics and group-level metrics of the study populations.

Group	Species	Group size	Males (proportion)
**Bonobo_F1**	Bonobo	6	0,50
**Bonobo_F2**	Bonobo	6	0,17
**Bonobo_LPZ**	Bonobo	9	0,56
**Bonobo_PLD**	Bonobo	9	0,33
**Bonobo_Lola3**	Bonobo	14	0,50
**Bonobo_Lola2**	Bonobo	17	0,59
**Bonobo_Lola1**	Bonobo	21	0,40
**ChimpB_LPZ**	Chimpanzee	6	0,17
**Chimp_Antwerp**	Chimpanzee	10	0,50
**Chimp_C3**	Chimpanzee	10	0,40
**Chimp_BB1**	Chimpanzee	12	0,42
**Chimp_BB2**	Chimpanzee	12	0,42
**Chimp_C4**	Chimpanzee	12	0,75
**ChimpA_LPZ**	Chimpanzee	17	0,35
**Chimp_C1**	Chimpanzee	22	0,33
**Chimp_C2**	Chimpanzee	42	0,38

References

1. Massen, J.J.M., and Koski, S.E. (2014). Chimps of a feather sit together: Chimpanzee friendships are based on homophily in personality. Evol. Hum. Behav. 35, 1–8. 10.1016/j.evolhumbehav.2013.08.008.
2. Verspeek, J., Staes, N., Leeuwen, E.J.C. van, Eens, M., and Stevens, J.M.G. (2019). Bonobo personality predicts friendship. Sci. Rep. 9, 19245. 10.1038/s41598-019-55884-3.
3. Stevens, J.M.G., De Groot, E., and Staes, N. (2015). Relationship quality in captive bonobo groups. Behaviour 152, 259–283. 10.1163/1568539X-00003259.
4. Samuni, L., Preis, A., Mielke, A., Deschner, T., Wittig, R.M., and Crockford, C. (2018). Social bonds facilitate cooperative resource sharing in wild chimpanzees. Proc. R. Soc. B Biol. Sci. 285, 20181643. 10.1098/rspb.2018.1643.
5. Thierry, B. (2007). Unity in diversity: Lessons from macaque societies. Evol. Anthropol. Issues, News, Rev. 16, 224–238. 10.1002/evan.20147.
6. DeTroy, S.E., Haun, D.B.M., and van Leeuwen, E.J.C. (2022). What isn't social tolerance? The past, present, and possible future of an overused term in the field of primatology. Evol. Anthropol. 31, 30–44. 10.1002/evan.21923.
7. Kaufhold, S.P., and van Leeuwen, E.J.C. (2019). Why intergroup variation matters for understanding behaviour. Biol. Lett. 15, 20190695. 10.1098/rsbl.2019.0695.
8. van Leeuwen, E.J.C., DeTroy, S.E., Kaufhold, S.P., Dubois, C., Schütte, S., Call, J., and Haun, D.B.M. (2021). Chimpanzees behave prosocially in a group-specific manner. Sci. Adv. 7, eabc7982. 10.1126/sciadv.abc7982.
9. Kaigaishi, Y., Nakamichi, M., and Yamada, K. (2019). High but not low tolerance populations of Japanese macaques solve a novel cooperative task. Primates 60, 421–430. 10.1007/s10329-019-00742-z.

10. van Leeuwen, E.J.C., Van Donink, S., Eens, M., and Stevens, J.M.G. (2021). Group-level variation in co-feeding tolerance between two sanctuary-housed communities of chimpanzees (Pan troglodytes). Ethology 127, 517–526. 10.1111/eth.13154.

11. van Leeuwen, E.J.C., Cronin, K.A., and Haun, D.B.M. (2018). Population-specific social dynamics in chimpanzees. Proc. Natl. Acad. Sci. U. S. A. 115, 11393–11400. 10.1073/pnas.1722614115.

12. Cronin, K.A., van Leeuwen, E.J.C., Vreeman, V., and Haun, D.B.M. (2014). Population-level variability in the social climates of four chimpanzee societies. Evol. Hum. Behav. 35, 389–396.

13. DeTroy, S.E., Ross, C.T., Cronin, K.A., van Leeuwen, E.J.C., and Haun, D.B.M. (2021). Cofeeding tolerance in chimpanzees depends on group composition: A longitudinal study across four communities. iScience 24, 102175. 10.1016/J.ISCI.2021.102175.
